# Health-Related Quality of Life in Patients with Arthritis: A Cross-Sectional Survey among Middle-Aged Adults in Chongqing, China

**DOI:** 10.3390/ijerph15040768

**Published:** 2018-04-16

**Authors:** Yunshuang Rao, Xianglong Xu, Dengyuan Liu, Cesar Reis, Ian M. Newman, Liqiang Qin, Manoj Sharma, Jun Shen, Yong Zhao

**Affiliations:** 1 School of Public Health and Management, Chongqing Medical University, No. 1 Yixueyuan Road, Yuzhong District, Chongqing 400016, China; rys0606@163.com (Y.R.); xianglong1989@126.com (X.X.); 18375750155@163.com (D.L.); 2 The Fourth Student Office of the School of Nursing, Chongqing Medical University, No. 1 Yixueyuan Road, Yuzhong District, Chongqing 400016, China; junshency@aliyun.com; 3 Research Center for Medicine and Social Development, Chongqing Medical University, Chongqing 400016, China; 4 The Innovation Center for Social Risk Governance in Health, Chongqing Medical University, Chongqing 400016, China; 5 Department of Preventive Medicine, Loma Linda University Medical Center, 24785 Stewart Street, Suite 204, Loma Linda, CA 92354, USA; cesarreis@hotmail.com; 6 Department of Educational Psychology, University of Nebraska-Lincoln, P.O. Box 880345, Lincoln, NE 68588, USA; inewman1@unl.edu; 7 School of Public Health, Soochow University, Suzhou 215000, China; qinliqiang@suda.edu.cn; 8 Department of Behavioral and Environmental Health, Jackson State University, Jackson, MS 39213, USA; manoj.sharma@jsums.edu; 9 School of Health Sciences, Walden University, 100 Washington Avenue South, Suite 900, Minneapolis, MS 55401, USA

**Keywords:** arthritis, quality of life, gender, lifestyle, adulthood

## Abstract

*Background:* Arthritis is a common disease in China, but few studies have been conducted to explore the associated health-related quality of life (HRQoL) and its influencing factors in Chongqing, China. This study aimed to explore the association of arthritis and HRQoL and probe factors affecting HRQoL among arthritis patients. *Methods:* A cross-sectional survey was conducted in Chongqing, China. A total of 1224 adults were included in the analysis. Medical Outcomes Study Short Form 36 Health Survey (SF-36) was used to measure HRQoL. Multiple linear regression models (stepwise) and covariance analysis models were used to examine the association of arthritis with HRQoL and analyze factors associated with HRQoL among arthritis patients. *Results:* Participants with arthritis had poorer HRQoL than those without. Among arthritis patients, the female was associated with a poorer state of physical functioning (*p* < 0.05); unemployed patients had a poorer state of role-physical than employed patients (*p* < 0.05); low average monthly income was associated with a poorer state of physical functioning (*p* < 0.01); childhood non-breastfeeding history was associated with a poorer state of social functioning (*p* < 0.01); average or dissatisfied attitude to current living conditions was associated with a poorer state of vitality and mental health (*p* < 0.05 for all); moreover, poor or general appetite was associated with a poorer state of role-physical, general health, social functioning, bodily pain, and role-emotional (*p* < 0.01 for all). *Conclusions:* Arthritis patients have worse HRQoL than those without in the Chinese population. Female, low socioeconomic status, childhood non-breastfeeding history, average or dissatisfied attitude to current living conditions and poor or general appetite were associated with poorer state of HRQoL among Chinese arthritis patients.

## 1. Introduction

Arthritis is a common disease that contributes to patients’ poor health status. Arthritis is the major cause of disability [1,2], a study conducted in 2013 found 58.48% of outpatients with arthritis had functional disability in southwest China [3]. Arthritis can affect joints and tissues and cause pain and stiffness [4]. Furthermore, arthritis can affect patients’ ability to accomplish daily activities, as well as their mobility, sleep duration, and level of social engagement [5]. The disease also contributes to substantial health-care costs [6]. In a study conducted from 2011 to 2012, the overall prevalence of arthritis among Chinese adults aged ≥45 years was 31.4% [7]. In the US, approximately 54.4 million (22.7%) adults were reported to have doctor-diagnosed arthritis during 2013–2015, among which 29.3% were in the 45–64 age group and 49.6% were in the 65 or older group [8].

Various factors were reported to be associated with the health-related quality of life (HRQoL). Health-related quality of life (HRQoL) refers to an individual’s or group’s perceived physical and mental health over time, it is a crucial component of health surveillance and considered to be a valid indicator of service needs and intervention outcomes [9]. This indicator is used to measure the aspect of quality of life that is influenced by arthritis and/or its treatment [10]. The HRQoL of arthritis patients is worse than those of healthy people [11,12]. Arthritis has a significant correlation with physical and emotional problems, adults with arthritis were significantly less likely to engage in physical activity than adults without arthritis [13], additionally, anxiety or depressions was significantly common among arthritis patients [14,15]. Previous studies have reported that low family income, inability to work, low social support, female gender, poor sleep quality, lower education level, and spinsterhood marital status were closely related to poor HRQoL in arthritis populations [11,12,16,17,18]. Our previous study found an association of hypertension with HRQoL [19]. Another study found obese and overweight adults had a poorer state of HRQoL than normal weight adults [20]. Unhealthy lifestyles were associated with worse emotional well-being [21]. Moreover, previous study proved decreased appetite was related to poor health status [22]. A China national representative survey in 2013 found that the weighted prevalence for ever having breastfed was 79.6% [23]. Breastfeeding has an impact on adult health, some previous studies probed the effect of breastfeeding, such as the effect on body composition, bone mass, and cardio respiratory risk factors and bone mass in adults [24,25,26], and the association between childhood breastfeeding history and HRQoL among hypertension patients was found in our previous study [19]. To our knowledge, our study is the first study to examine the association between breastfeeding history, healthy lifestyle, appetite, and HRQoL.

Findings on factors associated with HRQoL of arthritis patients have not been sufficiently clarified. In recent years, the Chinese government has paid much attention to the importance of lifestyle, breastfeeding, and health. The “Healthy China 2030” blueprint was released by the Chinese government in 2016 and the Chinese government issued the National Nutrition Program (2017–2030) in 2017 [27]. The prevalence of this disease increases significantly with age, the middle-aged and elderly are severely affected by arthritis [28] and since middle-aged adults are the predominant force in society, they undergo much distress and frustration in all aspects of life. Moreover, arthritis may influence their roles related to work and family and may even jeopardize their future professional plans [29]. Thus, clarifying the factors that affect HRQoL of arthritis patients is necessary to improve HRQoL of middle-aged arthritis patients in China. Therefore, this study has two research purposes: (1) examine the association of arthritis with HRQoL and (2) analyze factors associated with poor HRQoL among adults with arthritis.

## 2. Materials and Methods

### 2.1. Study Design and Sample

Detailed descriptions of the study design, a cross-sectional design was used, including the population sample, sampling framework, and pilot study have been previously reported [19,30]. The study was conducted in Chongqing city in July 2009. This study used a three-stage stratified random sampling method to recruit participants. Of 1250 questionnaires distributed, 1230 were returned and the number of valid responses after the exclusion of incomplete questionnaires was 1224, the response rate was 97.9% (1224/1250). Accordingly the prevalence of arthritis among middle-aged and older Chinese adults was 31.4% [9]. We set *P* = 0.314. “(*P* = 0.314; *Q* = 1 − *P* = 1 − 0.314 = 0.686 margin of error *d* = 0.1*P* = 0.1 × 0.314 = 0.0314, *Z_α_* = 1.96; the sampling size is $N=\frac{Z_{\alpha }^{2}\times pq}{d^{2}}=\frac{1.96^{2}\times 0.314\times 0.686}{0.0314^{2}}=896$. sampling size = 839).” Chongqing is located inland in the southwest region of China, it is one of China’s four directly controlled municipalities (the other three are Beijing, Shanghai, and Tianjin). By the end of 2016, the total number of permanent residents in Chongqing was 30.4843 million, the total population at the age of 0–14, 15–64, and over 65 years old is 5.0482 million (16.56%), 21.6164 million (70.91%), and 3.8197 million (12.53%), respectively. The total number of the male population is 15.4266 million (50.61%) and the number of the female population is 15.0577million (49.39%) [31].

### 2.2. Data Collection and Ethical Considerations

Investigators, who were specifically trained, were medical students from Chongqing. All investigators underwent standardized training and were familiar with the objectives and methodology. Medical students as investigators went back to their village or community to conduct the survey among local residents. As some of the participants had relatively low levels of education, we used face-to-face interview using a survey questionnaire to contribute to the participants understanding and completeness and thereby ensuring the quality of the survey. Participants were excluded who met the following exclusion criteria: cognitive disorders, including amnesia, dementia and delirium. Arthritis and other health conditions (hypertension) were assessed with questions such as: “Have you ever been told by a doctor or other health professional that you have been diagnosed with arthritis or hypertension?” All respondents were asked whether they had a previous diagnosis of arthritis or hypertension. If the answer was “yes,” then the participant was considered a patient with the condition. All of the participants were informed of the study’s purpose and their participation in the study was voluntary. This study was approved by the Ethics Committee of Chongqing Medical University (2014013). All participants provided written informed consent. All completed questionnaires were kept confidential. A unique identification number was assigned to each respondent.

### 2.3. Measurements

The questionnaire includes six sections: sociodemographic factors, childhood breastfeeding history, lifestyle factors, social support, appetite, and SF-36 questionnaire.

#### 2.3.1. Sociodemographic Factors

The first section determined the sociodemographic factors of the participants. Age was categorized as 45–47, 48–50, and 51–53 years old. Education level was categorized into three groups: low education (less than or equal to a primary and junior middle school education level), secondary education (more than or equal to a senior high school, including vocational/technical secondary school and junior college education level), and higher education (more than or equal to a senior college and university education level). Marital status was recorded as either married or cohabitation and living alone (including unmarried/divorced or separated/widowed). Average monthly income was classified as low (≤¥1601) and high (>¥1601), (1 USD = ¥6.34 in March 2018). Job condition was recorded as employed and unemployed. Self-reported height and weight were collected to calculate body mass index (BMI).

#### 2.3.2. Childhood Breastfeeding History

Breastfeeding history in childhood was also assessed and categorized as yes (breastfeeding) and no (non-breastfeeding, including artificial feeding or mixed feeding).

#### 2.3.3. Lifestyle Factors

Smoking and alcohol drinking status were recorded into two categories—yes (participants were current smokers/alcohol drinkers) or no (participants were not current smokers/alcohol drinkers). The frequency of self-reported regular physical activity and self-reported regular daily life (routine life) were categorized as usually and seldom/sometimes. Self-reported sleep quality was categorized as good and poor/average. Self-reported satisfaction with current living conditions (a summary of the recent life) were categorized as satisfied and average/not satisfied.

#### 2.3.4. Social Support

Self-reported perceptions of family relationship and self-reported relationship with colleagues or friends were classified as harmonious and poor/average.

#### 2.3.5. Appetite

Appetite was self-reported measured. Appetite in this study focuses on “desire to eat” and was categorized as good and poor or general.

#### 2.3.6. SF-36 Questionnaire

A validated Chinese version of the SF-36 questionnaire was used to measure participants’ HRQoL [32]. The questionnaire was widely adopted and the Cronbach’α of SF-36 questionnaire in this study is 0.76. To assess HRQoL, the SF-36 [33] has the following eight individual subscales: physical functioning (PF)—limitations in physical activities due to health concern, role-physical (RP)—role constraints due to physical health concern, bodily pain (BP), general health (GH)—general health perceptions, vitality (VT)—vigor and fatigue, social functioning (SF)—limitations in social activities due to health concern, role-emotional (RE)—role constraints due to emotional health concern and mental health (MH)—mental disorder and well-being. A low score under each subscale means a poor state under the corresponding subscale.

### 2.4. Statistical Analyses

Chi-square test or Fisher exact test was used to compare differences between categorical variables and the *t*-test was conducted to compare differences in continuous variables between arthritis and non-arthritis groups. We constructed the multiple linear regression model (stepwise) and the covariance analysis model [34] to examine the association of arthritis with HRQoL and analyze the factors associated with poor HRQoL among adults with arthritis. Variables based on previous studies were included in a multiple linear regression model (stepwise) among all participants, variables that have a *p*-value < 0.05 in the multiple linear regression model (stepwise) and two variables based on professional judgment (age and BMI) among all participants were included in the covariance analysis model among all participants, variables that have a *p*-value < 0.05 in the multiple linear regression model (stepwise) and two variables based on professional judgment (age and BMI) among all participants were included in the multiple linear regression model (stepwise) among arthritis patients and we included variables that have a *p*-value < 0.05 in the multiple linear regression model (stepwise) and two variables based on professional judgment (age and BMI)among arthritis patients in the covariance analysis model among arthritis patients. The data were analyzed using the Statistical Analysis System software (SAS version 9.1.3; SAS Institute, Cary, NC, USA).

## 3. Results

### 3.1. Characteristics of Participants According to Arthritis Status

A total of 1224 eligible respondents were investigated, including 165 (13.48%) patients with self-reported doctor-diagnosed arthritis and 1059 (86.52%) without arthritis. The average age of arthritis patients was 46.3 ± 2.6 years, whereas that of non-arthritis patients was 46.8 ± 2.7 years. Significant differences between the arthritis and non-arthritis groups were observed on educational level (*p* < 0.001), average monthly income (*p* < 0.001), sleep quality (*p* < 0.001), perception of family relationships (*p* = 0.004), relationships with colleague or friend (*p* < 0.001), satisfaction with current living conditions (*p* < 0.001) and appetite (*p* < 0.001) (see Table 1).

### 3.2. Score of Domains of SF-36 among All Participants According to Arthritis Status

The summary of the analysis on the scores of the SF-36 questionnaire among all participants showed that participants who suffer from arthritis are significantly lower in each domain of SF-36 (*p* < 0.001 for all). Male arthritis patients have a lower significant score in each domain of SF-36 (*p* < 0.005 for all). Female arthritis patients have significant lower score in seven domains of SF-36 (*p* < 0.05 for all), however, there was no significant difference in role-emotional between arthritis and non-arthritis among female participants (*p* = 0.097) (see Table 2).

### 3.3. Multiple Linear Regression Model for Factors Affecting HRQoL among All Participants

Multiple linear regression analysis among all participants found that gender was associated with poor state of physical functioning, general health, mental health, bodily pain, and role-emotional (*p* < 0.05 for all). Educational level was associated with good state of role-physical (*p* < 0.01). Marital status was associated with good state of all eight domains of SF-36 (*p* < 0.05 for all). Job condition was associated with good state of physical functioning, role-physical, vitality, social functioning, mental health, and role-emotional (*p* < 0.05 for all). Average monthly income was associated with good state of physical functioning (*p* < 0.05). Childhood breast feeding history was associated with good state of social functioning (*p* < 0.01). Alcohol drinking was associated with good state of vitality (*p* < 0.01). Regular physical activity was associated with good state of general health and vitality (*p* < 0.01 for all). Regular daily life was associated with poor state of general health, vitality and mental health (*p* < 0.05 for all). Sleep quality was associated with good state of physical functioning, vitality and mental health (*p* < 0.05 for all). Satisfaction with current living conditions was associated with good state of role-physical, bodily pain, general health, vitality, social functioning, role-emotional, and mental health (*p* < 0.01 for all). Perception of family relationships was associated with good state of social functioning, mental health, and bodily pain (*p* < 0.05 for all). Relationships with colleague or friend were associated with good state of role-physical, social functioning, mental health, and role-emotional (*p* < 0.05 for all). Appetite was associated with poor state of physical functioning, role-physical, bodily pain, general health, vitality, social functioning, and role-emotional (*p* < 0.05 for all). Hypertension was associated with poor state of physical functioning, role-physical, general health, vitality, social functioning (*p* < 0.05 for all). Arthritis was associated with poor state of physical functioning, role-physical, bodily pain, vitality, social functioning, role-emotional, and mental health (*p* < 0.05 for all) (see Table 3).

### 3.4. Covariance Analysis Model for Factors Affecting HRQoL among All Participants

Analysis of covariance among all participants found that compared with participants aged 51–53 years old, participants aged 48–50 years old had poorer state of social functioning and role-emotional (*p* < 0.05 for all). Male participants had better state of physical functioning, general health, mental health, and bodily pain than female participants (*p* < 0.05 for all). Compared with married or cohabitation participants, participants who were living alone had poorer state of all eight domains of SF-36 (*p* < 0.05 for all). Unemployed participants had poorer state of physical functioning, role-physical, vitality, social functioning, mental health and role-emotional than employed participants (*p* < 0.05 for all). Compared with participants whose average monthly income was more than ¥1601, participants whose average monthly income was no more than ¥1601 had poorer state of physical functioning (*p* < 0.05). Participants with childhood breastfeeding history had better state of social functioning than those without childhood breastfeeding history (*p* < 0.01). Compared with participants who drank alcohol, non-alcohol drinking participants had poorer state of vitality (*p* < 0.01). Compared with participants who usually engaged in regular physical activity, participants who seldom/sometimes engaged in regular physical activity had poorer state of general health and vitality (*p* < 0.01 for all). Compared with participants who usually had regular daily life, participants who seldom/sometimes had regular daily life had better state of general health, vitality, and mental health (*p* < 0.01 for all). Participants with poor/average sleep quality had poorer state of physical functioning, vitality and mental health than participants with good sleep quality (*p* < 0.01 for all). Compared with participants who were satisfied with current living conditions, participants who were general/not satisfied with current living conditions had poorer state of role-physical, general health, vitality, social functioning, mental health, bodily pain and role-emotional (*p* < 0.01 for all). Compared with participants who had harmonious perception of family relationships, participants who had bad/general perception of family relationships had poorer state of social functioning and bodily pain (*p* < 0.01 for all). Compared with participants who had poor/average relationships with colleague or friends, participants who had poor/general relationships with colleague or friends had poorer state of role-physical and role-emotional (*p* < 0.05 for all). Participants with good appetite had better state of physical functioning, role-physical, general health, vitality, social functioning, bodily pain and role-emotional than participants with poor or general appetite (*p* < 0.05 for all). Participants who had hypertension had poorer state of physical functioning, role-physical, general health, vitality, and social functioning than those without hypertension (*p* < 0.05 for all). Participants who had arthritis had poorer state in all eight domains of SF-36 than those without arthritis (*p* < 0.05 for all) (see Table 4).

### 3.5. Multiple Linear Regression Model for Factors Affecting HRQoL among Arthritis Participants

Multiple linear regression analysis among arthritis patients found that gender was associated with poor state of physical functioning and general health (*p* < 0.05 for all). Job conditions was associated with good state of physical functioning and role-physical (*p* < 0.05 for all). Average monthly income was associated with good state of physical functioning (*p* < 0.01). Childhood breastfeeding history was associated with good state of social functioning (*p* < 0.01). Sleep quality was associated with good state of mental health (*p* < 0.05). Satisfaction with current living conditions was associate with good state of mental health (*p* < 0.05). Appetite was associated with poor state of role-physical, social functioning, bodily pain and role-emotional (*p* < 0.05 for all). Hypertension was associated with poor state of physical functioning, general health and social functioning (*p* < 0.05 for all) (see Table 5).

### 3.6. Covariance Analysis Model for Factors Affecting HRQoL among Arthritis Participants

Analysis of covariance among arthritis patients found that male patients had better state of physical functioning than female patients (*p* < 0.05). Unemployed participants had poorer state of role-physical than employed patients (*p* < 0.05). Compared with arthritis participants whose average monthly income were more than ¥1601, participants whose average monthly income were no more than ¥1600 had poorer state of physical functioning (*p* < 0.01). Participants with higher BMI had better state of physical functioning (*p* < 0.05). Participants without childhood breastfeeding history had poorer state of social functioning than those with childhood breastfeeding history (*p* < 0.01). Compared with participants who usually had regular daily life, participants who seldom/sometimes had regular daily life had better state of vitality (*p* < 0.05). Compared with participants who were satisfied with current living conditions, participants who were average/not satisfied with current living conditions had poorer state of vitality and mental health (*p* < 0.05 for all). Participants with good appetite had better state of role-physical, general health, social functioning, bodily pain and role-emotional than participants with poor or general appetite (*p* < 0.01 for all). Participants with hypertension had poorer state of physical functioning, general health and social functioning than those without hypertension (*p* < 0.01 for all) (see Table 6).

## 4. Discussion

The study found that subjects with doctor-diagnosed arthritis have significantly worse HRQoL than those without arthritis in each domain of the SF-36 among Chinese middle-aged adults. Previous studies found that patients with arthritis experienced poorer HRQoL than those without arthritis [11,12]. This finding further confirms that arthritis significantly affects HRQoL among middle-aged Chinese adults. In view of the high prevalence of arthritis after 45 years old (31.4% in 2011–2012, China; 29.3% in 2013–2015, USA) [7,8], the findings from this study further hint that healthcare providers and public health practitioners should address the HRQoL of arthritis patients and targeted intervention to improve the HRQoL of patients with arthritis is necessary.

This study also found the role of arthritis on role-emotional in female participants was not significant. However, significant differences were observed in each domain of SF-36 among male participants. This suggested that while there was a decline in role-emotional, the middle-aged female arthritis participants did not think that they were emotionally affected. A previous study conducted in knee osteoarthritis patients among the elderly Taiwanese population found no significance in role-emotional among female participants, but a significant difference was found among male participants [35].

This study found that sociodemographic factors were associated with HRQoL among arthritis patients. Male arthritis participants have a higher score of physical functioning, which is consistent with previous study [12]. Compared with participants aged 51–53 years, participants aged 48–50 years had a poorer state of social functioning and role-emotional among all participants. However, no association was found between age and HRQoL among arthritis patients, we thought the possible reason may be that the age interval in each group (45–47 years old, 48–50 years old and 51–53 years old) is quite small. Association between age and HRQoL may be found in a larger age interval. We further found low socioeconomic status was positively associated with poor HRQoL among arthritis patients regarding socioeconomic determinants. This study found that unemployed patients had poorer state of role-physical than employed patients. One possible explanation for this finding may be that routine work distracted the employed patients from the physical discomfort caused by arthritis, thereby enabling them to adjust to their role better. A previous study showed that employment was positively associated with better HRQoL among Chinese patients with arthritis [12], our study addressed the role of employment in HRQoL among middle-aged arthritis patients. In addition, this study also found compared with participants who had a higher average monthly income, participants who had a lower average monthly income had a poorer state of physical functioning, which was in line with previous study [12]. Therefore, future intervention on improving HRQoL of arthritis patients should pay more attention to female arthritis patients and those with low socioeconomic status.

This study found that childhood breastfeeding history was associated with HRQoL among arthritis patients. Compared with arthritis participants with childhood non-breastfeeding history, arthritis participants with childhood breastfeeding history had a higher score of social functioning. The feeding pattern reflected nutrition after birth. A study conducted in Chinese women found that breastfeeding practice was associated with lower risk of arthritis [36]. A previous study showed that feeding patterns in infancy can influence cardiovascular development in childhood [37]. Our previous study found that hypertension respondents with breastfeeding history have better HRQoL than those without breastfeeding history [19]. This study stressed the importance of breastfeeding on HRQoL among arthritis patients. Clinical doctors and nurse can improve the HRQoL of arthritis patients by enhancing breastfeeding behavior. Also, this finding can provide some implications for implementation of Healthy China 2030 and the National Nutrition Program.

Unhealthy lifestyle was associated with HRQoL among arthritis patients in this study. The study found that compared with participants who usually had a regular daily life, participants who seldom/sometimes had a regular daily life had better state of vitality and no significant association was found between regular daily life and domains of SF-36 except vitality among those with arthritis. A possible reason for this may be that the quality of life assessment is subjective and influenced by a person’s life expectations [38]. We supposed that arthritis participants who seldom/sometimes had a regular daily life may have lowered their expectations and felt satisfied with a lower level of vitality. Future studies can further validate the relationship between the frequency of having a regular daily life and HRQoL among arthritis adults. Furthermore, we also found compared with participants who were satisfied with current living conditions, participants who were average/not satisfied with current living conditions had a poorer state of vitality and mental health. A study conducted in Eastern Europe found unhealthy lifestyles were associated with lack of information about health and behavior, which may have an impact on emotional well-being and contribute to poor health status [21].Hence, health care providers should focus on arthritis patients with unhealthy lifestyles and help them develop a healthy lifestyle and health educators should put more energy into health education regarding healthy lifestyle among arthritis patients.

This study found that arthritis participants with good appetite had better state of role-physical, general health, social functioning, bodily pain, and role-emotional than participants with a poor or general appetite. A study conducted among patients with heart failure found that appetite was associated with pain/discomfort and anxiety/depression [22]. Thus, health care providers and health professionals should pay a lot of attention to appetite and take measures to increase appetite among arthritis patients.

The present study has certain limitations that should be noted. First, recall bias is present in this study, for example, self-reported previous diagnosis of arthritis was obtained instead of a doctor’s diagnosis with regard to arthritis measurements. Self-reported arthritis may not be accurate because many people with arthritis may not be clear whether or not they have arthritis in China. Then, self-report of weight and height tends to be biased towards underestimating weight and overestimating height. Then, the self-reporting of childhood breastfeeding history has recall bias, while the duration of breastfeeding was not collected in this study. Lifestyle and social support are also self-reported, they also have recall bias. Self-reported indicators are subjective (such as childhood breast feeding history, and unhealthy lifestyle), objective indicators are needed to examine their association with HRQoL among arthritis patients in future studies. In addition, the instrument for measuring independent variables is self-designed without being validated. Second, cross-sectional survey data and insufficient sample reduced the ability of the researchers to perform direct causal inference to explore whether unmeasured factors may better explain the observed relationships and determine the direction of causality. Third, the participants are middle-aged (45–53 years old) and the findings may not be applicable to younger or older populations. Fourth, in this study, no strict distinction was made between the various types of arthritis. Fifth, the study is lack of justification for potential confounders (arthritis treatment and co-existing physical and mental conditions). Sixth, multiple chronic conditions may affect the HRQoL of arthritis populations. However, the study did not include multiple chronic conditions. Finally, we did not select menopausal information and former smoking information. Additionally, the investigators went back to their village or community to conduct the survey among local residents, which can itself cause selection bias.

## 5. Conclusions

Middle-aged arthritis patients have worse HRQoL than those without arthritis in China. Female, low socioeconomic status, childhood non-breastfeeding history, average or dissatisfied attitude to current living conditions and poor or general appetite were associated with worse HRQoL among middle-aged arthritis patients. This study provides detailed information that can help health care providers determine the HRQoL of patients with arthritis and then take measures to improve the HRQoL of the adults with arthritis.

## Figures and Tables

**Table 1:** Characteristics of participants according to arthritis status.

Characteristic	Non-Arthritis (*n* = 1059)	Arthritis (*n* = 165)	*p*-Value
**Sociodemographic Factors**			
**Gender (%)**			0.199
Male	602 (87.6%)	85 (12.4%)	
Female	457 (85.1%)	80 (14.9%)	
**Age (%)**			0.052
45–47 Years old	417 (88.7%)	53 (11.3%)	
48–50 Years old	395 (86.8%)	60 (13.2%)	
51–53 Years old	247 (82.6%)	52 (17.4%)	
**Educational Level (%)**			<0.001
Low education	142 (75.9%)	45 (24.1%)	
Secondary education	530 (86.9%)	80 (13.1%)	
Higher education	387 (90.6%)	40 (9.4%)	
**Marital Status (%)**			0.709
Living alone	81 (85.3%)	14 (14.7%)	
Married or cohabitation	978 (86.6%)	151 (13.4%)	
**Job Conditions (%)**			0.800
Unemployed	576 (86.7%)	88 (13.3%)	
Employed	483 (86.2%)	77 (13.8%)	
**Average Monthly Income (%)**			<0.001
≤1600 Yuan	581 (83.0%)	119 (17.0%)	
>1601 Yuan	478 (91.2%)	46 (8.8%)	
**Body Mass Index Mean(SD)**	23.4 (8.8)	22.7 (2.8)	0.075
**Childhood Breastfeeding History (%)**			0.328
No	197 (84.5%)	36 (15.5%)	
Yes	862 (87.0%)	129 (13.0%)	
**Lifestyle Factors**			
**Smoking (%)**			0.284
No	656 (87.4%)	95 (12.6%)	
Yes	403 (85.2%)	70 (14.8%)	
**Alcohol Drinking (%)**			0.193
No	468 (88.0%)	64 (12.0%)	
Yes	591 (85.4%)	101 (14.6%)	
**Regular Physical Activity (%)**			0.171
Seldom/Sometimes	809 (85.8%)	134 (14.2%)	
Usually	250 (89.0%)	31 (11.0%)	
**Regular Daily Life (%)**			0.276
Seldom/Sometimes	993 (86.8%)	151 (13.2%)	
Usually	66 (82.5%)	14 (17.5%)	
**Sleep Quality (%)**			<0.001
Poor/Average	607 (82.7%)	127 (17.3%)	
Good	452 (92.2%)	38 (7.8%)	
**Satisfaction with Current Living Conditions (%)**			<0.001
Unsatisfactory/Average	472 (81.5%)	107 (18.5%)	
Satisfactory	587 (91.0%)	58 (9.0%)	
**Social support**			
**Perception of Family Relationships (%)**			0.004
Poor/Average	242 (81.5%)	55 (18.5%)	
Harmonious	817 (88.1%)	110 (11.9%)	
**Relationships with Colleague or Friends (%)**			<0.001
Poor/Average	251 (79.9%)	63 (20.1%)	
Harmonious	808 (88.8%)	102 (11.2%)	
**Appetite (%)**			<0.001
Good	550 (91.2%)	53 (8.8%)	
Poor or general	509 (82.0%)	112 (18.0%)	
**Hypertension**			0.084
No	936 (87.2%)	138 (12.9%)	
Yes	123 (82.0%)	27 (18.0%)	

**Table 2:** Descriptive statistics of each domain of the SF-36 among Chinese adults according to arthritis status (mean, SD).

Domains	All Participants			Male			Female		
	Arthritis (*n* = 165)	Non-Arthritis (*n* = 1059)	*p*-Value	Arthritis	Non-Arthritis	*p*-Value	Arthritis	Non-Arthritis	*p*-Value
Physical functioning	79.2 (21.1)	88.5 (15.3)	<0.001	81.1 (21.7)	89.5 (15.3)	0.001	77.0 (20.4)	86.6 (15.9)	<0.001
Role-physical	76.4 (19.1)	86.0 (17.4)	<0.001	75.5 (19.0)	86.3 (17.3)	<0.001	77.3 (19.3)	85.0 (18.2)	0.001
Bodily pain	69.7 (18.7)	82.9 (17.2)	<0.001	70.3 (17.5)	84.0 (16.6)	<0.001	69.0 (19.9)	81.4 (17.8)	<0.001
General health	55.9 (15.1)	62.3 (14.4)	<0.001	57.3 (13.2)	62.8 (14.0)	0.001	54.1 (16.7)	61.6 (14.8)	<0.001
Vitality	59.9 (15.5)	66.1 (15.3)	<0.001	60.3 (14.6)	66.6 (15.1)	<0.001	59.5 (16.4)	65.4 (15.6)	0.002
Social functioning	76.5 (18.6)	85.4 (16.2)	<0.001	72.2 (16.2)	85.2 (16.2)	<0.001	79.5 (18.5)	84.9 (16.9)	0.010
Role-emotional	78.6 (18.3)	85.5 (16.5)	<0.001	76.9 (18.1)	85.8 (16.9)	<0.001	80.5 (18.4)	84.1 (17.6)	0.097
Mental health	63.1 (10.9)	67.4 (10.8)	<0.001	62.3 (10.5)	68.1 (10.6)	<0.001	63.6 (11.8)	66.4 (11.2)	0.041

**Table 3:** Multiple linear regression analysis for factors affecting health-related quality of life (HRQoL) among all participants in Chongqing, China.

Variable	PF	RP	GH	VT	SF	MH	BP	RE
	β (s.e.)	β (s.e.)	β (s.e.)	β (s.e.)	β (s.e.)	β (s.e.)	β (s.e.)	β (s.e.)
Age								
Gender	−4.5 (1.1) **	−1.8 (1.1)	−2.0 (0.9) *			−1.8 (0.7) **	−2.9 (1.1) *	–3.5 (1.3) **
Educational level		2.3 (0.8) **						
Marital status	7.8 (1.9) **	6.9 (2.0) **	4.2 (1.7) *	5.9 (1.7) **	5.0 (2.0) **	3.9 (1.3) **	7.1 (2.0) **	8.1 (2.0) **
Job conditions	4.8 (1.0) **	6.6 (1.1) **		2.1 (0.9) *	5.7 (1.0) **	1.5 (0.7) *		4.3 (1.1) **
Average monthly income	2.5 (1.1) *					−1.1 (0.7)		
Current BMI (mean, SD)							−0.1 (0.1)	−0.1 (0.1)
Childhood breast feeding history	2.4 (1.3)				4.7 (1.3) **		2.4 (1.4)	
Smoking								−2.5 (1.3)
Alcohol drinking				2.6 (1.0) **	−1.5 (1.1)			
Regular physical activity			3.3 (1.1) **	3.0 (1.1) **				
Regular daily life			−5.2 (2.0) **	−7.6 (2.0) **	−3.6 (2.2)	−3.7 (1.5) *		−3.9 (2.3)
Sleep quality	2.6 (1.2) *			3.1 (1.1) **		2.2 (0.8) **		
Satisfaction with current living conditions	2.1 (1.1)	3.7(1.2) **	3.6 (1.0) **	5.6 (1.0) **	3.3 (1.1) **	3.5 (0.8) **	3.3 (1.2) **	3.5 (1.2) **
Perception of family relationships					4.5 (1.4) **	1.9 (1.0) *	3.6 (1.4) **	2.9 (1.5)
Relationships with colleague or friends		3.0 (1.4) *	1.9 (1.1)		2.8 (1.4) *	2.0 (1.0) *		3.8 (1.5) **
Appetite	−2.9 (1.2) *	−4.1 (1.2) **	−3.6 (1.0) **	−2.8 (1.1) *	−3.5 (1.1) **		−3.0 (1.2) *	−3.5 (1.1) **
Hypertension	−8.1 (1.5) **	−3.8 (1.6) *	−5.6 (1.3) **	−3.5 (1.3) **	−3.3 (1.5) *			
Arthritis	−6.2 (1.5) **	−6.5 (1.6) **	−2.6 (1.3)	−3.1 (1.4) *	−6.5 (1.5) **	−2.8 (1.0) **	−12.2 (1.6) **	−3.8 (1.5) *
R^2^	0.1775	0.1726	0.1308	0.1752	0.1975	0.1414	0.1423	0.1556

Note: * with statistical difference (*p* < 0.05); ** with statistical difference (*p* < 0.01).

**Table 4:** Covariance analysis model for factors affecting HRQoL among all participants in Chongqing, China.

Variable	PF	RP	GH	VT	SF	MH	BP	RE
	β (s.e.)	β (s.e.)	β (s.e.)	β (s.e.)	β (s.e.)	β (s.e.)	β (s.e.)	β (s.e.)
45–47 Years old vs. 51–53 Years old	1.4 (1.3)	0.0 (1.4)	22,120.0 (1.2)	−1.7 (1.2)	0.6 (1.3)	−1.0 (0.9)	1.7 (1.4)	−1.4 (1.4)
48–50 Years old vs. 51–53 Years old	−0.2 (1.3)	−2.4 (1.4)	−1.5 (1.2)	−1.6 (1.2)	−2.7 (1.3) *	−0.9 (0.9)	0.4 (1.4)	−4.7 (1.4) **
Male vs. Female	4.3 (1.1) **		2.0 (0.9) *			1.7 (0.7) *	2.9 (1.1) **	1.9 (1.1)
Living alone vs. Married or cohabitation	−8.5 (1.9) **	−7.2 (2.0) **	−4.2 (1.7) *	−5.9 (1.7) **	−5.4 (1.9) **	−3.8 (1.3) **	−7.4 (2.0) **	−8.8 (2.0) **
Unemployed vs. Employed	−4.6 (1.0) **	−6.8 (1.1) **		−2.0 (0.9) *	−5.5 (1.0) **	−1.5 (0.7) *		−4.1 (1.1) **
≤¥1600 vs. >¥1601	−2.6 (1.1) *							
Current BMI (mean, SD)	0.0 (0.1)	−0.1 (0.1)	0.01 (0.1)	0.1 (0.1)	−0.0 (0.1)	−0.0 (0.0)	−0.1 (0.1)	−0.1 (0.1)
Childhood non-breastfeeding history vs. Childhood breastfeeding history					−4.7 (1.3) **			
Non-alcohol drinking vs. Alcohol drinking				−2.6 (1.0) **				
Seldom/Sometimes engage in regular physical activity vs. Usually engage in regular physical activity			−3.5 (1.1) **	−3.1 (1.1) **				
Seldom/Sometimes have a regular daily life vs. Usually have a regular daily life			5.5 (2.0) **	7.4 (2.0) **		3.6 (1.5) **		
Poor/Average sleep quality vs. Good sleep quality	−3.2 (1.2) **			−3.1 (1.1) **		−2.3 (0.8) **		
Average/Not satisfied with current living conditions vs. Satisfied with current living conditions		−3.7 (1.2) **	−4.0 (1.0) **	−5.5 (1.0) **	−3.5 (1.1) **	−3.3 (0.8) **	−3.4 (1.2) **	−4.0 (1.2) **
Poor/Average perception of family relationships vs. Harmonious perception of family relationships					−4.7 (1.4) **	−1.9 (1.0)	−3.6 (1.4) **	
Poor/Average relationships with colleague or friends vs. Harmonious relationships with colleague or friends		−3.3 (1.4) *			−2.6 (1.4)	−1.8 (1.0)		−5.0 (1.3) **
Good appetite vs. Poor or general appetite	3.2 (1.2) **	4.5 (1.2) **	3.8 (1.0) **	2.8 (1.1) *	3.4 (1.1) **		3.0 (1.2) *	3.9 (1.1) **
Non-hypertension vs. Hypertension	8.2 (1.5) **	3.4 (1.6) *	5.5 (1.3) **	3.8 (1.4) **	3.5 (1.5) *			
Non-arthritis vs. Arthritis	6.4 (1.5) **	7.1 (1.6) **	2.8 (1.3) *	3.2 (1.4) *	6.7 (1.5) **	2.7 (1.0) **	12.0 (1.6) **	4.3 (1.5) **
R^2^	0.17292	0.16835	0.13061	0.17873	0.20159	0.14148	0.14106	0.15783

Notes: PF: physical functioning; RP: role-physical; BP: bodily pain; GH: general health; VT: vitality; SF: social functioning; RE: role-emotional; MH: mental health; BMI: body mass index; * with statistical difference (*p* < 0.05); ** with statistical difference (*p* < 0.01).

**Table 5:** Multiple linear regression model for factors affecting HRQoL among arthritis participants in Chongqing, China.

Variable	PF	RP	GH	VT	SF	MH	BP	RE
	β (s.e.)	β (s.e.)	β (s.e.)	β (s.e.)	β (s.e.)	β (s.e.)	β (s.e.)	β (s.e.)
Age								
Gender	−7.2 (3.0) *		−5.2 (2.4) *			0.3 (1.7)	−2.9 (2.9)	1.7 (2.8)
Educational level		3.9 (2.0)						
Marital status	6.8 (5.5)	6.9 (5.0)	1.8 (4.2)	4.4 (4.3)	8.1 (4.9)	4.6 (3.0)	−1.0 (5.2)	8.7 (5.0)
Job conditions	7.0 (3.0) *	7.1 (2.8) *		2.6 (2.4)	1.8 (2.7)	1.5 (1.7)		2.5 (2.8)
Average monthly income	11.1 (3.3) **							
Current BMI (mean, SD)								
Childhood breastfeeding history					13.0 (3.3) **			
Smoking								
Alcohol drinking				3.3 (2.5)				
Regular physical activity			2.6 (2.9)	3.8 (3.0)				
Regular daily life			−7.6 (4.1)	−8.0 (4.2)		−5.2 (2.9)		
Sleep quality	6.4 (4.3)			3.8 (3.4)		4.2 (2.0) *		
Satisfaction with current living conditions		3.7 (3.3)	4.3 (2.6)	5.4 (2.6)	4.3 (3.1)	4.2 (1.9) *	4.7 (3.3)	4.7 (3.2)
Perception of family relationships					1.1 (3.3)	0.0 (2.1)	0.2 (3.3)	
Relationships with colleague or friends		−1.2 (3.2)			2.4 (3.4)	2.1 (2.1)		2.0 (3.2)
Appetite	−6.1 (3.9)	−10.9 (3.2) **	−5.0 (2.6)	−4.7 (3.1)	−7.5 (3.1) *		−8.4 (3.3) *	−7.1 (3.2) *
Hypertension	−16.6 (4.1) **	−6.7 (3.7)	−7.3 (3.2) *	−2.8 (3.1)	−9.3 (3.6) *			
R^2^	0.2524	0.2013	0.1418	0.1654	0.2753	0.1659	0.0756	0.1235

Notes: PF: physical functioning; RP: role-physical; BP: bodily pain; GH: general health; VT: vitality; SF: social functioning; RE: role-emotional; MH: mental health; BMI: body mass index; * with statistical difference (*p* < 0.05); ** with statistical difference (*p* < 0.01).

**Table 6:** Covariance analysis model for factors affecting HRQoL among arthritis participants in Chongqing, China.

Variable	PF	RP	GH	VT	SF	MH	BP	RE
	β (s.e.)	β (s.e.)	β (s.e.)	β (s.e.)	β (s.e.)	β (s.e.)	β (s.e.)	β (s.e.)
45–47 Years old vs. 51–53 Years old	−3.1 (4.3)	−1.8 (4.0)	−1.9 (3.1)	1.4 (3.4)	−1.9 (3.4)	−2.4 (2.3)	0.2 (4.0)	−7.0 (3.8)
48–50 Years old vs. 51–53 Years old	0.7 (4.2)	0.4 (4.0)	−1.8 (3.0)	4.8 (3.2)	−0.4 (3.3)	1.3 (2.3)	2.3 (4.0)	−4.1 (3.7)
Male vs. Female	8.9 (3.5) *		4.5 (2.6)					
Living alone vs. Married or cohabitation								
Unemployed vs. Employed	−6.0 (3.4)	−7.2 (3.2) *						
≤¥1600 vs. >¥1601	−12.6 (3.7) **							
Current BMI (mean, SD)	1.3 (0.6) *	0.8 (0.6)	−0.0 (0.4)	0.4 (0.5)	0.8 (0.5)	0.0 (0.3)	0.3 (0.6)	0.5 (0.6)
Childhood non-breastfeeding history vs. Childhood breastfeeding history					−19.6 (3.5) **			
Non-alcohol drinking vs. Alcohol drinking								
Seldom/Sometimes engage in regular physical activity vs. Usually engage in regular physical activity								
Seldom/Sometimes have a regular daily life vs. Usually have a regular daily life				10.8 (5.2) *				
Poor/Average sleep quality vs. Good sleep quality						−4.1 (2.2)		
Average/Not satisfied with current living conditions vs. Satisfied with current living conditions				−5.5 (2.7) *		−4.6 (1.9) *		
Poor/Average perception of family relationships vs. Harmonious perception of family relationships								
Poor/Aeverage relationships with colleague or friends vs. Harmonious relationships with colleague or friends								
Good appetite vs. Poor or general appetite		13.1 (3.5) **	9.7 (2.7) **		9.9 (3.0) **		11.7 (3.5) **	13.4 (3.3) **
Non-hypertension vs. Hypertension	19.2 (4.2) **		9.3 (3.1) **		9.4 (3.4) **			
R^2^	0.26221	0.1696	0.17288	0.09856	0.37068	0.10602	0.09343	0.15156

Notes: PF: physical functioning; RP: role-physical; BP: bodily pain; GH: general health; VT: vitality; SF: social functioning; RE: role-emotional; MH: mental health; BMI: body mass index; * with statistical difference (*p* < 0.05); ** with statistical difference (*p* < 0.01).
